# Cholinergic neurons in the basal forebrain are involved in behavioral abnormalities associated with Cul3 deficiency: Role of prefrontal cortex projections in cognitive deficits

**DOI:** 10.1038/s41398-023-02306-8

**Published:** 2023-01-24

**Authors:** Maximiliano Rapanelli, Wei Wang, Edward Hurley, Maria Laura Feltri, Christopher Pittenger, Luciana Romina Frick, Zhen Yan

**Affiliations:** 1 Department of Physiology and Biophysics, Jacobs School of Medicine, University at Buffalo, The State University of New York, Buffalo, USA; 2 Department of Neurology, Jacobs School of Medicine, University at Buffalo, The State University of New York, Buffalo, USA; 3 Institute for Myelin and Glia Exploration, University at Buffalo, The State University of New York, Buffalo, USA; 4 Department of Biochemistry, Jacobs School of Medicine, University at Buffalo, The State University of New York, Buffalo, USA; 5 Neuroscience Graduate Program. Jacobs School of Medicine, University at Buffalo, The State University of New York, Buffalo, USA; 6 Departments of Psychiatry and Psychology, Yale Child Study Center, and Interdepartmental Neuroscience Program, Yale University School of Medicine, Buffalo, USA; 7 Department of Medicine, Jacobs School of Medicine, University at Buffalo, The State University of New York, Buffalo, USA; 8 Clinical and Translational Research Center, Jacobs School of Medicine, University at Buffalo, The State University of New York, Buffalo, USA

**Keywords:** Autism spectrum disorders, Molecular neuroscience

## Abstract

Loss-of-function mutations of the gene Cul3 have been identified as a risk factor for autism-spectrum disorder (ASD), but the pathogenic mechanisms are not well understood. Conditional Cul3 ablation in cholinergic neurons of mice (Chat^CRE^Cul3^F/+^) recapitulated ASD-like social and sensory gating phenotypes and caused significant cognitive impairments, with diminished activity of cholinergic neurons in the basal forebrain (BF). Chemogenetic inhibition of BF cholinergic neurons in healthy mice induced similar social and cognitive deficits. Conversely, chemogenetic stimulation of BF cholinergic neurons in Chat^CRE^Cul3^F/+^ mice reversed abnormalities in sensory gating and cognition. Cortical hypofunction was also found after ChAT-specific Cul3 ablation and stimulation of cholinergic projections from the BF to the prefrontal cortex (PFC) mitigated cognitive deficits. Overall, we demonstrate that cholinergic dysfunction due to Cul3 deficiency is involved in ASD-like behavioral abnormalities, and that BF cholinergic neurons are particularly critical for cognitive component through their projections to the PFC.

## Introduction

Autism spectrum disorder (ASD) is a developmental neuropsychiatric condition characterized by social deficits and stereotypic behaviors, sometimes accompanied by intellectual disability [1]. ASD is considered a polygenic disorder, with mutations, duplications and deletions in multiple genes [2, 3]. A few of them have been associated with high risk for this disorder. Among the most strongly implicated genes is Cul3 [4, 5], in which recurrent *de novo* loss-of-function mutations have been identified in several large genetic screenings [6–11]. Cul3 encodes a core component of the E3 ubiquitin ligase complex, which recognizes protein substrates that need to be degraded and recruits the E2 ubiquitin-conjugating enzyme [12, 13]. How Cul3 mutations contribute to the pathophysiology of ASD is not well understood. We previously generated a forebrain-restricted Cul3 conditional knockout mouse using Emx1-Cre recombination; this mouse recapitulates core behavioral abnormalities associated with autism [14]. However, loss of Cul3 in these mice was limited to glutamatergic neurons in the forebrain, and the model did not recapitulate the intellectual disabilities observed in patients with autism [15, 16]. Therefore, we hypothesize that Cul3 in other neurons may also contribute to the pathophysiology of ASD.

Striatal interneurons, including those expressing choline acetyl transferase (ChAT), were shown to elude Emx1-Cre driven recombination [17]. The core behavioral abnormalities in autism are anatomically linked to cortico-striatal abnormalities [18]. Most acetylcholine (ACh) in the circuitry comes from such striatal interneurons that act locally upon medium spiny neurons of the striatum (STR) or from projection neurons in the basal forebrain (BF) that send their axons to multiple regions, such as the prefrontal cortex (PFC), hippocampus, thalamus and midbrain [19, 20]. ACh from these neurons modulates cognition, attention, cue detection, goal-oriented behaviors, and reward processing [21–24].

Pre-clinical and clinical studies have provided converging evidence of dysfunctional cholinergic neurotransmission in ASD. Proton magnetic resonance studies in ASD patients have shown reduced choline and an impairment in cholinergic neurotransmission that correlates with symptom severity [25–27]. ASD patients also exhibit lower expression of M1 muscarinic acetylcholine receptors in various cortical regions [28, 29]. Additionally, reduction in the expression levels of different nicotinic acetylcholine receptor subunits has been described in the thalamus of patients with ASD [30]. Several clinical studies suggest that the acetylcholinesterase inhibitors rivastigmine, galantamine, and donepezil, alone or in combination with choline, can relieve some of the symptoms in autistic children [31–34].

Animal studies have shed light on the possible contribution of cholinergic neurotransmission to core autistic behaviors. Social motivation is modulated by ACh, and it is impaired by cholinergic denervation of neocortical inputs in rats [35, 36]. M4(Gi) muscarinic receptor knockout mice develop social interaction abnormalities, hyperlocomotion, and pre-pulse inhibition (PPI) deficits [37]. M1(Gq) muscarinic receptor knockout mice display increased social contact, as well as hyperlocomotion [38, 39]. Knockout of the nicotinic receptor β2 subunit increases social contact, which can be normalized by re-expression of this receptor in the PFC [40]. Administration of nicotine or donepezil improves sociability and decreases repetitive behaviors in the BTBR mouse idiopathic model of autism-like behaviors [41, 42]. Nevertheless, these models lack construct validity, and abnormalities of cholinergic neurotransmission are not directly linked to the etiology and pathophysiology of ASD. Here, we developed a new mouse that relies on ChAT^+^ cell-specific Cul3 deletion and studied the differential contribution of cholinergic neurons in the BF and STR to autism-associated behaviors and cognition.

## Material and methods

### Animals

All procedures were approved and supervised by the Institutional Animal Care and Use Committee (University at Buffalo). Cul3^flox/fox^ mice (https://www.jax.org/strain/028349) were bred with Chat-ires-Cre mice (https://www.jax.org/strain/006410) to generate cholinergic neuron-specific Cul3 knockout mice. Animals were housed in groups of four, with food and water ad libitum, with controlled humidity and temperature (22–23 °C) in a 12/12 h light/dark cycle. Both female and male mice were used in this study. Mice were 4 weeks old at the time of surgery. Behavioral testing, electrophysiological recording or tissue harvesting were performed when mice were 6 weeks old. No randomization was used given that the experimental group was assigned by the mouse’s genotype. All experiments were conducted at least twice, and all replicates are shown.

### Stereotaxic virus injection

Mice were deeply anesthetized with ketamine/xylazine (100 mg/kg; 10 mg/kg) and placed in a mouse stereotaxic frame (Stoelting, USA). A 10 µl syringe (7000 series, Hamilton, USA) attached to a micropump was lowered through to skull burr hole into the PFC (AP + 1.8 mm, L ± 0.3 mm, DV −2.7 mm), STR (AP + 1 mm, L ± 1.5 mm, DV −2.7 mm) or BF (AP = + 0.26,ML: ± 1,DV = 5.4). Mice were injected with the following viruses: AAV5-hSyn-DIO-hM3D(Gq)-mCherry, AAV5-hSyn-DIO-hM4D(Gi)-mCherry, or rg-AAV-hSyn-DIO-hM3D(Gq)-mCherry viruses (≥ 7 × 10^12^ vg/ml, Addgene) within the PFC (0.3 µl per hemisphere), STR (0.5 µl per hemisphere) or BF (0.5 µl per hemisphere) at a flow rate of 50 nl/min. These constructs have been thoroughly validated by us and others in terms of their efficiency at recombining and activating/inactivating neurons [43–46]. Clozapine N-oxide (CNO) was injected intraperitoneally at a dose of 3 mg/kg in sterile saline solution 1 hour prior to the behavioral procedures. The response to CNO has been previously shown to be specific, as compared to Cre negative or fluorophore-only injected animals [44, 46].

### Behavioral assays

#### Social preference test

Three-chamber social interaction test was performed as previously described [14]. Briefly, an apparatus containing 3 chambers with manually retractable doorways allowing for access to side chambers was used (L: 101.6 cm, W: 50.8 cm, H: 50.8 cm). The test consisted of 2 phases with different stimulus in each of two side chambers. The stimulus was placed inside a capsule (an inverted mesh pencil cup, D: 10.2 cm, H: 10.5 cm). During habituation, mice freely explored the apparatus with two empty capsules for 10 min. In the 1^st^ phase of testing, two identical nonsocial stimuli (paper ball) were used. In the 2^nd^ phase of testing, a nonsocial stimulus (wood block) and a social stimulus (matched mouse in strain, sex and age) were used. The test animal was placed in the center of the chamber and allowed to explore the apparatus for 10 min. Interaction time was counted based on close “investigating” behaviors of the test animal to each stimulus. Preference index was calculated as: (Social Investigation Time - Non-social Investigation Time) / Total Time. ANY-maze software (Stoelting, USA) was used for automated quantification.

#### Locomotion and open field

Mice were placed in an arena (L: 101.6 cm, W: 50.8 cm, H: 50.8 cm) and their overall activity was recorded for 30 minutes. The time spent in the center area (L: 71.6 cm, W: 33 cm) was also quantified.

#### Rotarod test

Motor coordination was measured in an accelerating rotarod (SD instruments, San Diego CA). Mice were placed in the apparatus, which slowly accelerated from 4 to 40 rpm over a 5‐min test session, and the falling time was recorded.

#### Grooming

Mice were individually placed in a clean cage with 1 cm of bedding to avoid other behaviors to interfere. After 20 min of habituation, cumulative grooming during a 10-minute time window was quantified manually blind to genotype/treatment.

#### Startle response and PPI

All testing was performed in the startle response system SR-LAB (San Diego Instruments, USA). The session started with a 5 min habituation period, followed by four consecutive blocks of test trials. Background (67 dB) noise was present during the entire course of the experiment. There were 8 different types of trials in one session. “Pre-pulse” trials consisted of a 20 ms pre-pulse of 70 dB, 76 dB, or 85 dB that preceded the 120 dB (20 ms) startle pulse by 100 ms (onset to onset). “Startle” trials consisted of a startle stimulus of a 40 ms pulse of 90 dB, 100 dB, 110 dB or 120 dB, whereas the “no stimulus” trials measure the basal movement of the mice with background noise. A session consisted of 74 trials of randomized trials separated by an average time of 14 sec (range: 8–20 s). PPI was calculated as: (pulse alone – prepulse)/pulse alone.

#### Barnes maze

Briefly, the mouse was placed on a circular platform with eight equally spaced holes at the edge, one of which was attached with an escape box. As an aversive stimulus to promote escape from the platform, we used a strong light. During the information acquisition (learning) phase (two pre-tests), the animal was allowed to explore the platform until finding the correct hole using distal visual cues and entering the escape box. The interval between each of the learning phase was 5 min. After performance in two learning sessions, the mouse was placed in its home cage to rest for 15 min. During the information retention and retrieval (memory) phase (one test), the escape box was removed, and the animal was put back on the platform to explore for 5 min. The time spent on the correct hole (T1) and the other seven incorrect holes (T2) were counted. Spatial memory index was calculated as T1/T2 [47].

#### Temporal order recognition memory

The temporal order recognition memory (TORM) task was performed as previously described [48]. This paradigm has been validated by us and others to assess memory in rats and mice [49–52]. For habituation, the test mouse was placed into a rectangular enclosure (L: 48.2 cm, W: 38.1 cm, H: 25.4 cm) with opaque walls for 3 min and then returned to its home cage for 5 min. Then, the mouse was exposed to two identical objects (object 1) placed in opposing corners of the enclosure for 5 min. After a 1 h break, the mouse was exposed to another two identical objects (object 2) for 5 min. After a three-hour break, mice were then exposed to one copy of each object for 5 min. The amount of time spent investigating each object was recorded and used to calculate a discrimination ratio: Time on novel (less recent) object 1 – Time on familiar (more recent) object 2 / Time on both objects.

### Electron microscopy

Sciatic nerves were fixed in 2% buffered glutaraldehyde, then post-fixed in 1% osmium tetroxide. After alcohol dehydration, nerves were sequentially submerged in propylene oxide, Epon:propylene oxide (1:1 mixture), and 100% Epon. Resin was allowed to polymerize. Ultrathin transverse sections were sliced 700–900Å-thick using a Leica UC7 microtome, stained with uranyl acetate and lead citrate, and examined with a FEI BioTwin electron microscope as described [53].

### Electrophysiological recording

Mouse brain slices (300 µm) were positioned in a perfusion chamber attached to the fixed stage of an upright microscope (Olympus) and submerged in continuously flowing oxygenated (95% O_2_ and 5% CO_2_) low-Mg^2+^ ACSF to slightly elevate the basal neuronal activity (in mM: 130 NaCl, 26 NaHCO_3_, 1 CaCl_2_, 0.5 MgCl_2_, 3.5 KCl, 1.25 NaH_2_PO_4_, 10 glucose, pH 7.4, 300 mOsm). Cells were visualized with a water-immersion lens (40×) and a CCD camera. A Multiclamp 700 A amplifier with Clampex 8.2 software and Digidata1322A (Molecular Devices) was used for recordings. Recording pipettes were pulled from glass capillaries (1.5 mm OD and 0.86 mm ID) with resistance at 3–5 MΩ by a pipette puller (Model P-97, Sutter Instrument Co.). For spontaneous action potential (sAP) recordings in BF cholinergic neurons, the internal solution contained (in mM: 20 KCl, 100 K-gluconate, 10 HEPES, 4 ATP, 0.5 GTP, and 10 phosphocreatine). A small depolarizing current was applied to adjust the inter-spike potential at −55 mV to −58 mV. Electrophysiological data were analyzed with Clampfit 10.0.7 (Molecular Devices) and Mini Analysis 6.0.3 (Synaptosoft).

### Immunofluorescence and confocal microscopy

Mice were transcardially perfused with 4% paraformaldehyde (pH: 7.4). Brains were sliced (30 µm) using a vibratome (Leica, USA). Slices were blocked for 1 h at RT in PBS with 0.3% Triton X-100 and 5% donkey serum. Then, slices were incubated with goat anti-ChAT antibody (1:1000 AB144P Millipore-Sigma), rabbit anti-phospho rpS6 S235/S236 (Cell Signaling; #4858 S, 1:500), rabbit anti-phospho rpS6 S240/S244 (Cell Signaling; #5364 P, 1:500), or rabbit anti-Arc (1:1000, Abcam, ab23382) overnight at 4 °C. After washing, slices were incubated with an Alexa Fluor 568-labeled donkey anti-rabbit secondary antibody (1:400, A10042, Life Technologies), washed again and mounted with Vectashield (Vectorlabs, USA). Confocal imaging was performed in a Zeiss LSM 510 microscope (Zeiss, Germany). Manual counting was conducted blind to genotype. Signal intensity quantification was performed with ImageJ software.

### Statistical analysis

Sample size is specified in the figure legend for each experiment and was previously calculated G*Power software v3.1. All values are expressed as mean ± SEM. Exclusion criteria was value outside two standard deviations from mean for a 95% confidence interval. Statistical analyses were performed using GraphPad Prism. T-test, One-Way ANOVA, Two-way ANOVA, or repeated measures ANOVA with Tukey’s or Šidák’s post-hoc tests were used depending on the experimental design. Normal distribution and variances between groups were tested for each parameter investigated. Degrees of freedom (Dfn, DFd or df) and F values are shown in figure legends. All comparisons were considered significant if *P* < 0.05.

## Results

### Cul3 deficiency in cholinergic neurons causes gross anatomical abnormalities

**Fig. 1:**
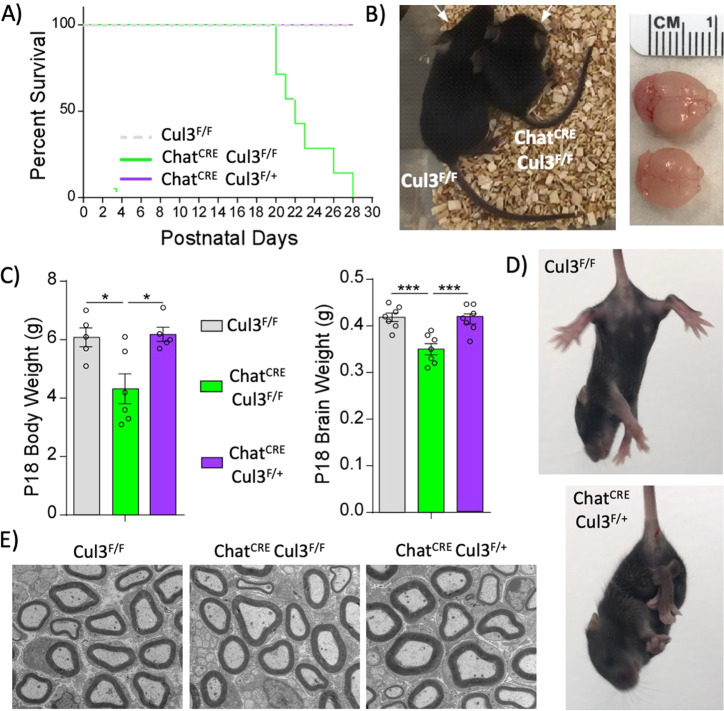
Early postnatal effects of Cul3 deficiency in cholinergic neurons. **A** Kaplan-Meier survival curve of the different genotypes showing the postnatal lethality of mice caused by biallelic Cul3 knockout in cholinergic neurons (χ^2^(2) = 38.51, *p* < 0.001; Cul3^F/F^: *n* = 16, Chat^CRE^Cul3^F/F^: *n* = 13, Chat^CRE^Cul3^F/+^: *n* = 17). **B** Representative images of visual differences in body and brain sizes between a Cul3^F/F^ mouse and a Chat^CRE^Cul3^F/F^ sibling. **C** Differences in body (left) and brain (right) weight across the genotypes at postnatal day p18 (Body weight: F(2,13) = 7.18, *p* = 0.008, one-way ANOVA, Cul3^F/F^: *n* = 5, Chat^CRE^Cul3^F/F^: *n* = 6, Chat^CRE^Cul3^F/+^: *n* = 5; Brain weight: F(2,18) = 13.94, *p* = 0.0002, one-way ANOVA, *n* = 7 mice/genotype). Tukey’s post-hoc test was used for multiple comparisons. *p < 0.05; ***p < 0.001. **D** Clenching phenotype in Chat^CRE^Cul3^F/+^ mice (bottom) that is absent in wild-type littermates. **E** Representative pictures of transmission electron microscopy of sciatic nerves across the different genotypes, showing no evident abnormalities.

To generate a cholinergic neuron-specific Cul3 knockout, we crossed Chat-IRES-Cre mice with Cul3 floxed mice. Homozygous mice (Chat^CRE^Cul3^F/F^) with deletion of both Cul3 alleles in cholinergic neurons were viable during embryonic and early postnatal stages, but their survival began to decline around postnatal day p21, with 100% lethality by p28 (Fig. 1A). These mice not only had smaller body size (Fig. 1B) and body weight (Fig. 1C), but also a significant reduction in their brain size and weight (Fig. 1B, C). On the other hand, haploinsufficient mice lacking one copy of Cul3 in cholinergic neurons (Chat^CRE^Cul3^F/+^) had no reduced viability, survival, or weight in comparison to Cul3^F/F^ mice (Fig. 1). Although Chat^CRE^Cul3^F/+^ mice display a limb clenching phenotype, motor neurons in their peripheral nerves are healthy, without any obvious signs of axonal abnormalities or neurodegeneration (Fig. 1D, E). Taking these observations into account, the following studies were performed in Chat^CRE^Cul3^F/+^ mice and compared to wild-type (Cre negative) littermates.

### Cul3 deficiency in cholinergic neurons recapitulates behavioral abnormalities of autism

**Fig. 2:**
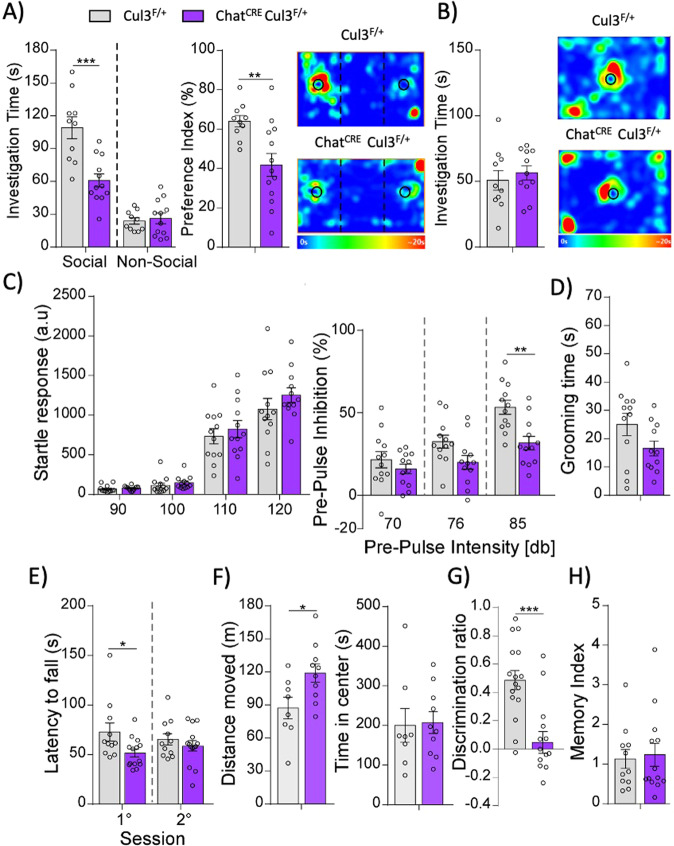
Behavioral abnormalities after Cul3 ablation in cholinergic neurons. **A** Social preference deficits in Chat^CRE^Cul3^F/+^ mice demonstrated by reduced social interaction time and social preference index in 3-chamber sociability tests (Cul3^F/+^: *n* = 10, Chat^CRE^Cul3^F/+^: *n* = 13, Time: F_interaction_(1,40)=16.11, *p* = 0.0003, F_zone_(1,40)=90.36, *p* < 0.0001, F_genotype_(1,40)=13.18, *p* = 0.0008, two-way ANOVA; Index: *t*(21) = 3.14, *p* = 0.005, unpaired two-tailed t-test). **B** Normal social approach behavior in Chat^CRE^Cul3^F/+^ mice (Cul3^F/+^: *n* = 10, Chat^CRE^Cul3^F/+^: *n* = 11, *t*(19) = 0.63, *p* = 0.54, unpaired two-tailed t-test). **C** Chat^CRE^Cul3^F/+^ mice show normal startle response at increasing startle stimulus (left) (Cul3^F/+^: *n* = 12, Chat^CRE^Cul3^F/+^: *n* = 12, F_interaction_(3,66) = 0.53, *p* = 0.74, F_stimulus_(3,66) = 148.4, *p* < 0.0001, F_genotype_(1,22) = 0.88, *p* = 0.36, two-way RM ANOVA), but impaired PPI after increasing pre-pulse intensities (right) (F_interaction_(2,44) = 4.88, *p* = 0.012, F_stimulus_(2,44) = 44.24, *p* < 0.0001, F_genotype_(1,22) = 7.1, *p* = 0.01, two-way RM ANOVA). **D** Grooming time as an indicator of repetitive behaviors is not affected in Chat^CRE^Cul3^F/+^ mice (Cul3^F/+^: *n* = 12, Chat^CRE^Cul3^F/+^: *n* = 11, *t*(21) = 1.76, *p* = 0.093, unpaired two-tailed t-test). **E** Motor coordination in rotarod tests is mildly impaired in Chat^CRE^Cul3^F/+^ mice (Cul3^F/+^: *n* = 11, Chat^CRE^Cul3^F/+^: *n* = 14, F_interaction_(1, 46) = 1.49^,^ *p* = 0.23, F_session_(1, 46) = 0.0003, *p* = 0.99, F_genotype_(1, 46) = 5.3, *p* = 0.026, two-way RM ANOVA). **F** Chat^CRE^Cul3^F/+^mice display hyperlocomotion and no indication of anxiety-like behaviors (Cul3^F/+^: *n* = 8, Chat^CRE^Cul3^F/+^: *n* = 10, Distance moved: *t*(16) = 2.45, *p* = 0.026; Time in center: *t*(16) = 0.1421, *p* = 0.89, unpaired two-tailed *t*-test). **G** Temporal order recognition memory (TORM) deficits in Chat^CRE^Cul3^F/+^ mice (Cul3^F/+^: *n* = 16, Chat^CRE^Cul3^F/+^: *n* = 14, *t*(28) = 4.31, *p* = 0.0002, unpaired two-tailed t-test). **H** Barnes maze spatial memory is normal in Chat^CRE^Cul3^F/+^ mice (Cul3^F/+^: *n* = 11, Chat^CRE^Cul3^F/+^: *n* = 13, t(22) = 0.28, *p* = 0.78, unpaired two-tailed t-test). Tukey’s and Šidák’s tests were used for multiple comparisons. *p < 0.05; **p < 0.01; ***p < 0.001.

We evaluated the behavioral effects of Cul3 haploinsufficiency in cholinergic neurons on autism-like behaviors, cognition, and motor function. First, we analyzed the performance of Chat^CRE^Cul3^F/+^ and control mice in a three-chamber social preference test, which is a well validated paradigm to measure sociability in rodents [14, 54, 55]. Chat^CRE^Cul3^F/+^ mice had the reduced interaction time and preference index for the social stimulus over the non-social stimulus (Fig. 2A), indicating social preference deficits. Cul3 deficiency in cholinergic neurons did not affect social approach behavior (Fig. 2B).

Sensory gating mediated by cortico-striatal circuits is measured by PPI; deficient PPI has been observed in patients with various neuropsychiatric diagnoses including ASD [56]. Since cholinergic neurons play a critical role in modulating cortico-striatal circuits, we evaluated PPI in Chat^CRE^Cul3^F/+^ and wild-type mice. Loss of Cul3 in cholinergic neurons did not affect basal startle response, but Chat^CRE^Cul3^F/+^ mice showed significant PPI deficits across prepulse intensities, most markedly at the highest intensity (85db; Fig. 2C).

Dysfunction of cholinergic interneurons in the STR could lead to motor coordination and locomotion abnormalities. To test repetitive behaviors in Chat^CRE^Cul3^F/+^ mice, we quantified basal grooming as previously described [14, 44, 55] and found no differences between genotypes (Fig. 2D). Chat^CRE^Cul3^F/+^ mice showed mild to insignificant changes in the latency to fall in the accelerating rotarod test (Fig. 2E), indicating overall normal motor coordination, consistent with healthy peripheral cholinergic neurons found in sciatic nerves (Fig. 1E). When tested in an open field, Chat^CRE^Cul3^F/+^ mice showed mild hyperlocomotion and unchanged amount of time spent in the center (Fig. 2F). Nevertheless, the total distance traveled in the 3-chamber social preference test was not different between genotypes, indicating that the social impairment is not due to abnormal locomotion (Supplementary Fig. 1).

Since cholinergic neurotransmission is important for cognition, attention, and memory [24, 57, 58], we tested Chat^CRE^Cul3^F/+^ mice in a temporal order object recognition task (TORM), which examines the animal’s ability to differentiate between familiar objects presented previously at different times. Chat^CRE^Cul3^F/+^ mice displayed severe cognitive deficits, compared to control animals in the TORM task despite their similar total exploration time (Fig. 2G, Supplementary Fig. 2). BF cholinergic neurons project to the hippocampus, and dysfunction of the cholinergic neurotransmission can affect spatial memory and learning [57, 59, 60]. Thus, we examined the performance Chat^CRE^Cul3^F/+^ mice in the Barnes maze task, which tests the animal’s ability to recall the location of a hole that was previously attached to an escape box, among seven other incorrect holes. Chat^CRE^Cul3^F/+^ mice did not exhibit any deficits in spatial memory in the Barnes maze task (Fig. 2H).

### Cul3 regulates the activity of BF cholinergic neurons

**Fig. 3:**
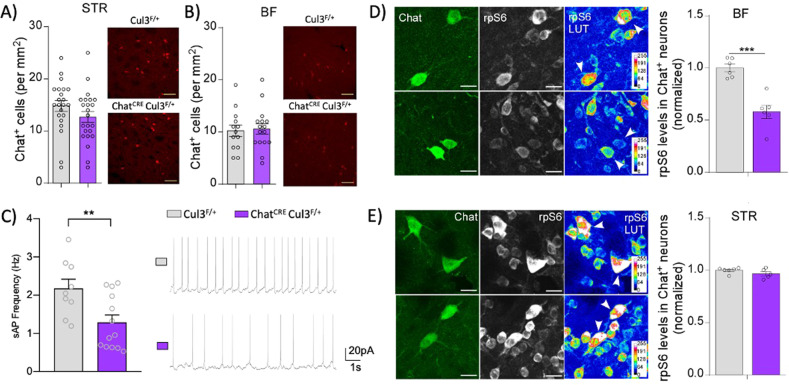
Reduced activity of BF cholinergic neurons by Cul3 ablation. **A**, **B** Unchanged numbers of cholinergic neurons in both STR and BF of Chat^CRE^Cul3^F/+^ mice (STR: *n* = 22 slices/6 Cul3^F/+^ mice, *n* = 23 slices/6 Chat^CRE^Cul3^F/+^ mice, t(43) = 1.43, *p* = 0.16; BF: *n* = 13 slices/4 Cul3^F/+^ mice, *n* = 16 slices/4 Chat^CRE^Cul3^F/+^ mice, t(27) = 0.22, *p* = 0.83, unpaired two-tailed t-test). **C** Plot of synaptic-driven, spontaneous action potential (sAP) frequencies showing the significant reduction in BF cholinergic neurons of Chat^CRE^Cul3^F/+^ mice (*n* = 9 cells/3 Cul3^F/+^ mice, *n* = 13 cells/4 Chat^CRE^Cul3^F/+^ mice, t(20) = 2.85, *p* = 0.0098, unpaired two-tailed t-test). Inset: Representative sAP traces. **D**, **E** Representative confocal images and quantification of rpS6-S240/S244 in ChAT^+^ cholinergic neurons in the BF (**D**) or the STR (**E**) (**D**, *n* = 70 cells/6 Cul3^F/+^ mice, *n* = 67 cells/6 Chat^CRE^Cul3^F/+^ mice, t(10) = 5.69, *p* = 0.002; **E**, *n* = 68 cells/6 Cul3^F/+^ mice, *n* = 54 cells/5 Chat^CRE^Cul3^F/+^ mice, t(9) = 1.42, *p* = 0.19, unpaired two-tailed t-test). In all figures, **p < 0.01, ***p < 0.001.

We next investigated how the ablation of Cul3 in cholinergic neurons affects their activity and properties. The total number of cholinergic neurons in Chat^CRE^Cul3^F/+^ and wild-type mice was similar in both STR and BF (Fig. 3A, B), indicating that there is no overall cholinergic neuron loss. To evaluate whether Cul3 deficiency induces dysfunction in cholinergic neurotransmission, we measured the activity of BF cholinergic neurons, identified by morphology (size and shape) and electrophysiological properties. The frequency of synaptically-driven, spontaneous action potentials was strongly reduced in BF cholinergic neurons of Chat^CRE^Cul3^F/+^ mice (Fig. 3C). We also quantified the levels of rpS6-S240/244, which correlate with cholinergic activity, [61–64] in BF cholinergic neurons by confocal imaging. We found that the levels of rpS6-S240/244 were significantly reduced in BF cholinergic neurons from Chat^CRE^Cul3^F/+^ mice relative to Cul3^F/+^ controls (Fig. 3D), but not in STR cholinergic neurons (Fig. 3E). These electrophysiological and imaging data demonstrate that deletion of Cul3 in cholinergic neurons substantially reduces their activity, specifically in the basal forebrain.

### BF cholinergic neuron activity is critical for autism-linked behaviors and cognition

Our findings provide evidence that Cul3 deletion in cholinergic neurons both affects their firing rate in the BF and generates loss of social preference and cognitive deficits. We hypothesized that dysfunction of BF cholinergic neurons may be causative of the observed cognitive and behavioral phenotypes. Therefore, we tested the necessity and sufficiency of cholinergic neurons in the BF to generate such abnormalities.

**Fig. 4:**
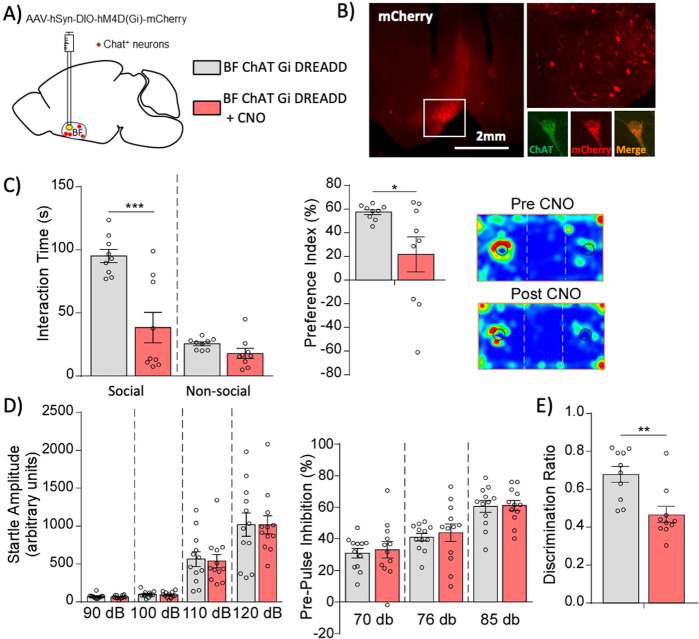
Chemogenetic inhibition of BF cholinergic neurons in healthy animals induces social and cognitive deficits. **A** Schematic representation of stereotaxic delivery of Gi-coupled hM4D inhibitory floxed DREADD AAV (mCherry-tagged) into the basal forebrain of Chat^CRE^ mice. **B** mCherry signal in the BF confirming recombination (upper right: zoomed in image, lower right: individual cholinergic neuron). **C** Induction of social preference deficits after inactivation of BF cholinergic neurons of control mice after CNO administration (*n* = 9, Interaction time: F_interaction_(1, 32) = 12.56, *p* = 0.0012, F_zone_(1,32) = 42.27, *p* < 0.0001, F_treatment_(1,32) = 21.58, *p* = 0.0001, two-way ANOVA; Preference index: t(16) = 2.40, *p* = 0.029, unpaired two-tailed t-test). Inset: Representative heat maps. **D** Startle response and sensory gating processing are not affected by BF cholinergic neuron inhibition (*n* = 12, Startle: F_interaction_(3, 66) = 0.014, *p* = 1.0; F_stimulus_(3, 66) = 86.67, *p* < 0.0001; F_treatment_(1, 22) = 0.017, *p* = 0.90; PPI: F_interaction_(2, 44) = 0.014, *p* = 0.09; F_stimulus_(2, 44) = 57.12, *p* < 0.0001; F_treatment_(1, 22) = 0.16, *p* = 0.70, two-way RM ANOVA). **E** Reduced discrimination ratio in the temporal order recognition memory (TORM) test after inhibiting BF cholinergic neurons (*n* = 10, t(18) = 3.58, *p* = 0.0021, unpaired two-tailed t-test). Šidák’s post-hoc test was used for multiple comparisons. In all panels, *p < 0.05; **p < 0.01; ***p < 0.001.

We first tested whether inactivation of BF cholinergic neurons in otherwise normal animals is able to induce similar behavioral phenotypes. An inhibitory double floxed Gi-coupled hM4D DREADD AAV fused with mCherry under the control of human synapsin promoter (AAV5-hSyn-DIO-hM4D(Gi)-mCherry) was stereotaxically injected into the BF of Chat^CRE^ mice (Fig. 4A, B). This strategy ensures recombination is restricted to BF cholinergic neurons. Then, mice were tested in the aforementioned battery of behavioral procedures before and after CNO injection, which activates the DREADD receptors and thus inhibits the BF cholinergic neurons. Inhibition of BF cholinergic neurons impaired social preference, as shown by the reduced interaction time with the social stimulus and the reduced social preference index (Fig. 4C, Supplementary Fig. 1), without significant effects on PPI (Fig. 4D). Cognitive performance on the TORM task was also impaired after inactivation of BF cholinergic neurons (Fig. 4E, Supplementary Fig. 2).

We further examined the impact of chemogenetic inactivation of cholinergic neurons in the STR of healthy mice. No significant alterations were found in the behavioral assays, including social preference, TORM and PPI despite the efficient infection and recombination (Supplementary Fig. 3).

**Fig. 5:**
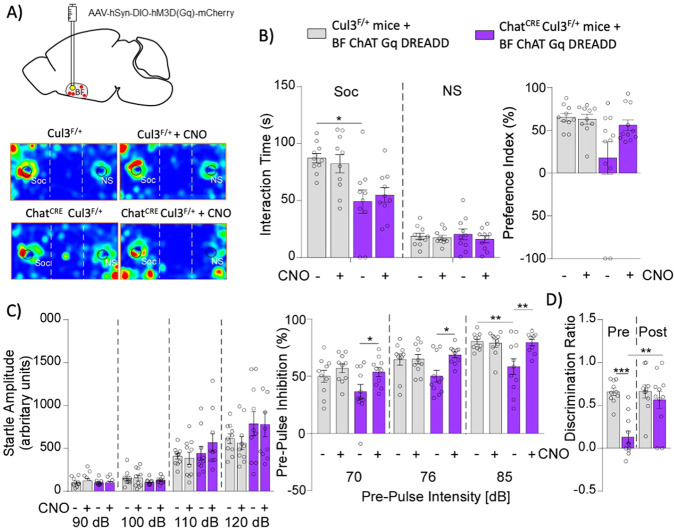
Chemogenetic activation of BF cholinergic neurons in Chat^CRE^Cul3^F/+^ mice ameliorates the PPI and cognitive abnormalities. **A** Schematic representation of AAV-hM3D(Gq) DREADD injection into the BF of Chat^CRE^Cul3^F/+^ mice. **B** Unchanged social interaction time in 3-chamber social preference tests after CNO activation of BF cholinergic neurons in Chat^CRE^Cul3^F/+^ mice (Cul3^F/+^: *n* = 10, Chat^CRE^Cul3^F/+^: *n* = 11, interaction time: F_interaction_(3, 76) = 2.96, *p* = 0.037; F_zone_(1, 76) = 125.1, *p* < 0.0001; F_treatment_(3, 76) = 2.23, *p* = 0.09; preference index: F_interaction_(1, 38) = 3.39, *p* = 0.073; F_genotype_(1, 38) = 6.2, *p* = 0.017, F_treatment_(1, 38) = 2.68, *p* = 0.11; two-way ANOVA). **C** PPI deficit is ameliorated by CNO activation of BF cholinergic neurons in Chat^CRE^Cul3^F/+^ mice (Cul3^F/+^: *n* = 10, Chat^CRE^Cul3^F/+^: *n* = 11, Startle: F_interaction_(9, 108) = 1.54, *p* = 0.14, F_stimulus_(3, 108) = 98.94, *p* < 0.0001, F_genotype/treatment_(3, 36) = 0.66, *p* = 0.58. PPI: F_interaction_(6, 74) = 0.90, *p* = 0.5, F_stimulus_(2, 74) = 87.82, *p* < 0.0001, F_genotype/treatment_(3, 37) = 5.2, *p* = 0.0041, two-way RM ANOVA). **D** TORM impairments is reversed by CNO activation of BF cholinergic neurons in Chat^CRE^Cul3^F/+^ mice (Cul3^F/+^: *n* = 10, Chat^CRE^Cul3^F/+^: *n* = 11, F_interaction_(1, 38) = 7.92, *p* = 0.0077; F_genotype_(1, 38) = 16.73, *p* = 0.0002; F_genotype/treatment_(1,38) = 8.25, *p* = 0.0066, two-way ANOVA, Tukey’s and Šidák’s post-hoc tests were used for multiple comparisons). In all figures, **p < 0.01; ***p < 0.001.

Next, we evaluated whether activation of BF cholinergic neurons from Chat^CRE^Cul3^F/+^ mice can rescue the behavioral abnormalities. To achieve this, an excitatory Gq-coupled hM3D (AAV5-hSyn-DIO-hM3D(Gq)-mCherry) DREADD was delivered into the BF of these mice (Fig. 5A). Although a non-significant trend towards an improvement in the preference index was observed, the interaction time with social stimuli remained low after CNO injection in Chat^CRE^Cul3^F/+^ mice, (Fig. 5B), indicating that activation of BF cholinergic neurons failed to rescue social deficits. In contrast, the PPI and TORM deficits in Chat^CRE^Cul3^F/+^ mice were ameliorated by chemogenetic stimulation of the activity of BF cholinergic neurons (Fig. 5C, D). Altogether, these results suggest that cholinergic activity in the BF is critical for at least some of the autism-associated behavioral changes.

### BF cholinergic innervation to PFC is key to cognitive deficits in Chat^CRE^Cul3^F/+^ mice

**Fig. 6:**
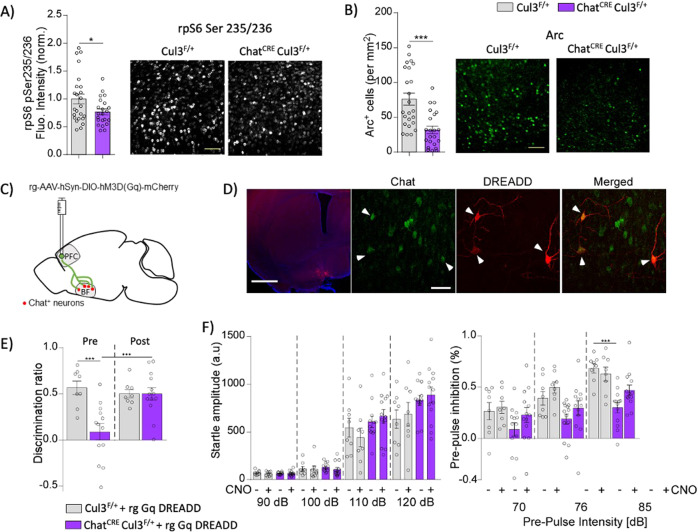
Activation of BF cholinergic neurons that project to the PFC reverses cognitive deficits in Chat^CRE^Cul3^F/+^ mice. **A**, **B** Hypoactivation of cortical neurons as measured by reduced levels of rpS6-Ser235/236 (**A**) and decreased number of Arc^+^ cells (**B**) in the PFC of Chat^CRE^Cul3^F/+^ mice, compared to Cul3^F/+^ littermates (*n* = 24 slices/6 Cul3^F/+^ mice, *n* = 22 slices/6 Chat^CRE^Cul3^F/+^ mice, **A**, t(44) = 2.16, *p* = 0.036, **B**, t(44) = 4.25, *p* < 0.0001, unpaired two-tailed t-test). Scale bar 40 µm. **C** Schematic representation of stereotaxic delivery of floxed retrograde hM3D(Gq) DREADD AAV into the PFC of Chat^CRE^Cul3^F/+^ mice. **D** Confocal images showing DREADD mCherry in the BF (left) and Cre-dependent recombination in ChAT^+^ neurons (right). Scale bar: left 1 mm, right 20 µm. **E** Improved cognitive performance in the TORM test of Chat^CRE^Cul3^F/+^ mice after CNO administration to activate the cholinergic BF to PFC pathway (Cul3^F/+^: *n* = 8, Chat^CRE^Cul3^F/+^: *n* = 13, F_interaction_(1, 38) = 9.107, *p* = 0.0045; F_genotype_(1, 38) = 9.211, *p* = 0.0043; F_treatment_(1,38) = 4.887, *p* = 0.0332, two-way ANOVA). **F** PPI deficits in Chat^CRE^Cul3^F/+^ mice are unchanged by CNO administration (Startle: F_interaction_(9, 111) = 1.75, *p* = 0.086, F_stimulus_(3, 111) = 184.9, *p* < 0.0001, F_treatment_(3, 37) = 1.48, *p* = 0.24. PPI: F_interaction_(6, 76) = 3.33, *p* = 0.0057, F_stimulus_(2,76) = 105.7, *p* < 0.0001, F_treatment_(3, 38) = 4.78, *p* = 0.0064; two-way RM ANOVA, Tukey’s post-hoc test was used for multiple comparisons). In all figures, *p < 0.05; ***p < 0.001.

Cholinergic neurons in the BF project to the PFC. We studied the consequences of BF cholinergic dysfunction in the PFC of Chat^CRE^Cul3^F/+^ mice by measuring two cellular markers that are activated by ACh: rpS6-Ser235/236, a readout of mTOR activation, [55, 65–69] and the immediate early gene Arc, which has been linked to synaptic plasticity and neuronal activity. Quantitative immunohistochemical analysis showed a significant reduction of rpS6-Ser235/236 and Arc levels in the PFC of Chat^CRE^Cul3^F/+^ mice (Fig. 6A, B), suggesting that Cul3 deficiency in the cholinergic system induced PFC hypoactivity.

To establish whether social and cognitive deficits in Chat^CRE^Cul3^F/+^ mice may be caused, at least in part, by the decreased cortical neuron activity as a result of the reduced BF cholinergic projection, we delivered a retrograde rg-AAV-hSyn-DIO-hM3D(Gq)-mCherry (rg-Gq) DREADD virus into the PFC. Through retrograde transportation, the floxed DREADD virus recombines at the BF in ChAT-Cre expressing neurons that project to the PFC [70] (Fig. 6C, D). After CNO administration to activate BF cholinergic to PFC pathway, the cognitive deficits in TORM tests of Chat^CRE^Cul3^F/+^ mice were significantly improved (Fig. 6E, Supplementary Fig. 2), while sensory gating was not affected (Fig. 6F).

## Discussion

Here we establish a link between the ASD high-risk gene *Cul3* and the cholinergic system, showing that Cul3 ablation in cholinergic neurons impairs their activity while producing deficits in social interaction, sensory-gating, and cognition – all traits of relevance to the symptoms of ASD. Moreover, we demonstrated that cholinergic projections from the BF to the PFC are implicated in the cognitive component of the Cul3 phenotype (summarized in Supplementary Table 1).

Previous reports showed that full Cul3 knockout is embryonically lethal but Cul3 haploinsufficient mice are viable [71–73]. Similarly, ChAT homozygous null pups (but not heterozygous mice) die at birth [64, 74, 75], suggesting that ACh is also critical for proper development. Biallelic deletion of Cul3 in cholinergic neurons causes size and weight reductions of the body and brain, and death within a month after birth. On the other hand, ChAT^+^-specific Cul3 heterozygous mice are viable and overall healthy, with normal body and brain weight and size and without any gross anatomic abnormalities. There were no obvious signs of neurodegeneration in the peripheral nervous system where motor neurons express ChAT, suggesting that neurodevelopmental defects may be circumscribed to the brain. Moreover, limb clasping has been observed in other ASD models and attributed to cerebellar dysfunction [71, 76, 77]. These lines of evidence suggest that Cul3 expression in cholinergic neurons is necessary for proper development of the central nervous system.

Cholinergic neurons are implicated in a number of behavioral outputs [24, 36, 78–80]. Here, we focused on autism-relevant behaviors and cognitive tests. Monoallelic Cul3 ablation in ChAT^+^ neurons recapitulated social preference deficits similar to those seen in mice with Cul3 deficiency in forebrain glutamatergic neurons and other Cul3 models of autism [14, 43, 72]. ChAT-Cre driven Cul3 ablation also caused an impairment of PPI, similar to Emx1-Cre driven Cul3 ablation [14], suggesting that alterations in the cholinergic tone may underlie the sensory-gating processing defects. Interestingly, some similar behavioral deficits have been observed in other mice with cholinergic dysfunction [81, 82].

In addition to these abnormalities, Cul3 ablation in cholinergic neurons caused a profound impairment in temporal order object recognition memory; this may model cognitive deficits frequently observed in ASD [15]. Similarly, heterozygous null (Cul3^+/-^) mice have a reduced preference for the novel object in the novel object recognition test [72] and exhibit impaired cognitive flexibility after contextual fear conditioning [71]. GFAP-Cre driven deletion of Cul3 does not affect spatial working memory in the Y maze test [43]. Consistently, we observed normal performance in the Barnes maze spatial memory test. Thus, Cul3 may be implicated in only a subset of cognitive functions.

Cul3 deficiency reduced the activity of BF cholinergic neurons. Thus, we characterized their contribution to behavioral deficits in Chat^CRE^Cul3^F/+^ mice. Chemogenetic inhibition of BF cholinergic neurons recapitulated the social and cognitive deficits seen in Chat^CRE^Cul3^F/+^ mice but did not affect PPI, indicating that cholinergic tone from the BF is necessary for proper cognition and social interaction. On the other hand, inhibition of STR cholinergic interneurons did not reproduce any of the behavioral abnormalities, which is in line with their normal rpS6 levels. Chemogenetic stimulation of BF cholinergic neurons from Chat^CRE^Cul3^F/+^ mice reversed the cognitive and sensorimotor gating deficits but not the social impairments. We speculate that cholinergic dysfunction during neurodevelopment may produce long-term deleterious effects on brain regions critical for social behaviors, such as the PFC [35], providing a possible explanation on why chemogenetic stimulation of BF neurons could not rescue the social deficits. Our findings suggest that the activity of BF cholinergic neurons is necessary for cognitive performance in the TORM task and that reduced activity in these cells explains the deficits observed in Chat^CRE^Cul3^F/+^ mice.

Our findings also suggest that cholinergic interneurons in the STR may play a minor or negligible role in these phenotypes. It is possible that Cul3 is involved in the functioning of other types of interneurons. Parvalbumin (PV) fast-spiking GABAergic interneurons have been implicated in the pathophysiology of ASD [83]. For example, reduced numbers of PV^+^ interneurons were found in the STR of Shank mice [84]. Moreover, PV knockout mice display behavioral phenotypes with relevance to all three core symptoms present in human ASD patients: abnormal reciprocal social interactions, impairments in communication and repetitive and stereotyped patterns of behavior [85]. One proposed pathophysiological mechanism of PV interneurons in ASD is an excitation/inhibition circuit imbalance. Maternal loss of the ubiquitin-protein ligase E3A gene (Ube3a) associated with Angelman syndrome produces an excitatory/inhibitory imbalance through neuron type-specific synaptic defects, causing inhibitory deficits from fast-spiking interneurons in the neocortex [86]. Insufficiency of another member of the cullin family, Cul4B, has been reported to decrease the number of parvalbumin PV^+^ interneurons in the dentate gyrus, and has been linked to X-linked mental retardation [87]. Given that Cul3^+/-^ mice have reduced numbers of cortical PV^+^ interneurons [71], Cul3 loss may similarly affect PV interneurons playing a role in ASD-like behaviors; this possibility needs to be investigated.

At a circuit level, cholinergic neurons in the BF project to and strongly modulate the PFC [24]. The PFC is a key brain region for cognition, attention and social behaviors [21, 23, 35, 40], and PFC dysfunction is associated with autistic like behaviors and cognitive deficits [14, 54, 88, 89]. Our data suggests that BF cholinergic neurons are sufficient and necessary for TORM task performance. Because cholinergic modulation of the PFC is involved in object recognition memory [90], we selected this region among all brain areas innervated by the BF. The PFC of Chat^CRE^Cul3^F/+^ mice displayed a downregulation of neuronal rpS6-S235/236 and Arc, indicating PFC hypofunction, which is likely caused by a reduction of the cholinergic influence. Our findings are supported by previous evidence showing that stimulation of cholinergic neurons in the BF by DREADDS can induce activation of cortical neurons [91]. The tight control that cholinergic neurons exert over the cortex prompted us to speculate that the impaired BF cholinergic projection to PFC is, at least partially, responsible for the behavioral phenotypes in Chat^CRE^Cul3^F/+^ mice. Chemogenetic stimulation of BF cholinergic to PFC pathway significantly improved cognitive deficits in Chat^CRE^Cul3^F/+^ mice. While the role of BF cholinergic neurons in cognition has been previously demonstrated [92], our data is the first one to relate it to a high-risk gene for ASD, connecting it to those neurons that project specifically to the PFC. Given that CNO was administered systemically, we cannot rule out that this treatment did not activate cholinergic neurons in other brain regions that also innervate the PFC. The PFC receives some fibers from the pedunculopontine nucleus and the laterodorsal tegmental area -although the functional significance of this is unknown- but it has been demonstrated that most cholinergic axons originate from the basal forebrain [93]. Thus, it is more likely that the behavioral phenotype after retrograde chemogenetic activation is the result of the ACh input coming from the BF.

Strikingly, while chemogenetic inhibition of BF cholinergic neurons had no effect on sensorimotor gating, activation of these neurons in Chat^CRE^Cul3^F/+^ mice reversed the PPI deficits. One explanation for the lack of effect of BF-specific silencing of cholinergic tone may be the multi-regional regulation of sensory gating. It is plausible that concomitant cholinergic inhibition of other brain regions is needed to affect PPI. Nevertheless, our data suggests that activation of BF neurons is sufficient to alleviate PPI deficits. Given that retrograde stimulation of cholinergic projections to the PFC also improves PPI deficits, we can speculate that cortical ChAT interneurons are affected in Chat^CRE^Cul3^F/+^ mice perhaps lowering ACh levels locally and subsequently triggering the sensorimotor gating defects, but stimulation of BF projection neurons may elevate cortical ACh contributing towards PPI deficit alleviation.

In summary, deficiency of the ASD high risk gene Cul3 in cholinergic neurons renders them dysfunctional, which in turn triggers the impairment of social and cognitive behaviors. Cholinergic neurons residing in the BF were found to be key for these abnormalities, and their projection to the PFC is particularly important for cognition.

## Supplementary information

Suppl table and figures
