# Supplementary material for: Cholinergic neurons in the basal forebrain are involved in behavioral abnormalities associated with Cul3 deficiency: Role of prefrontal cortex projections in cognitive deficits
Source: Transl Psychiatry. 2023 Jan 24;13:22. doi: 10.1038/s41398-023-02306-8 (PMC9873627; doi:10.1038/s41398-023-02306-8)
Supplement: Supplementary file 1 — Suppl table and figures [file 41398_2023_2306_MOESM1_ESM.docx]

**Supplementary figure 1**

**
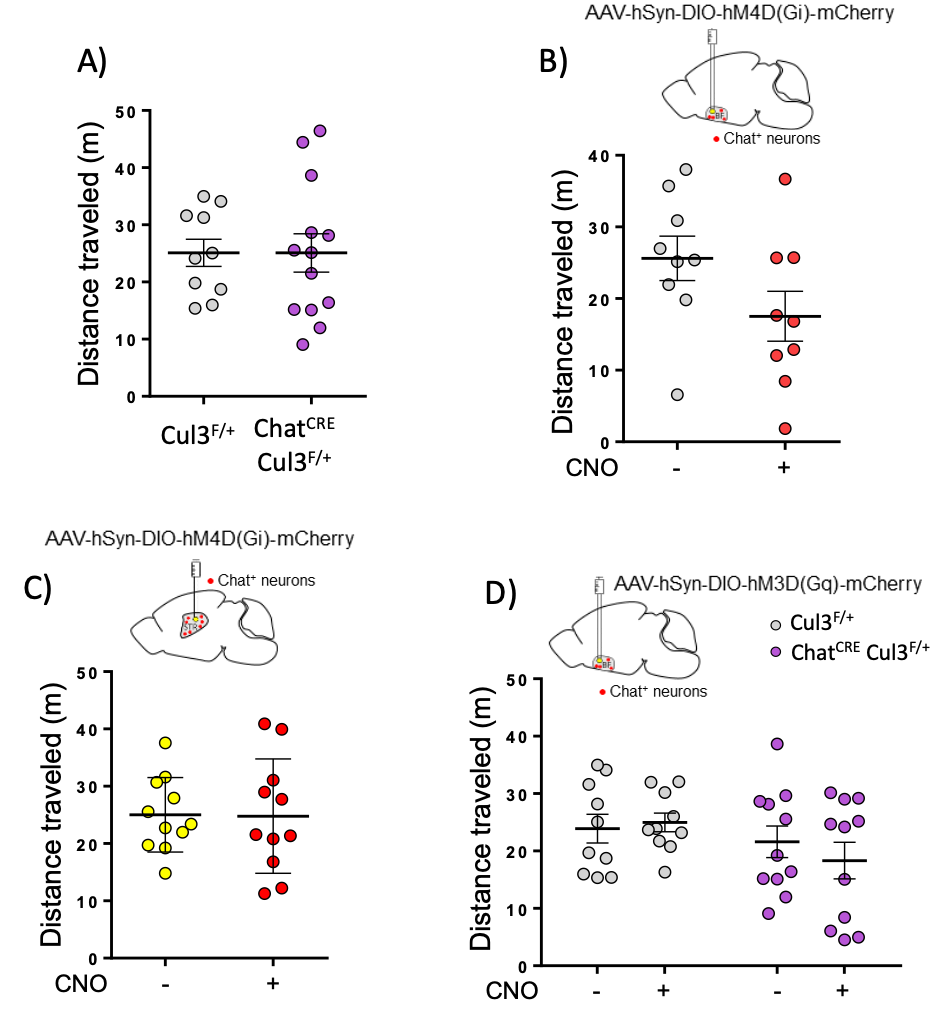
**

**Supplementary Figure 1. Locomotor activity during the social preference test.** No significant differences were observed in the total distance traveled by the mouse during the 3-chamber social preference between **A)** Chat^CRE^Cul3^F/+^ and Cul3^F/+^ mice (Cul3^F/+^: n=10, Chat^CRE^Cul3^F/+^: n=13, t(21)=0.003, p=0.99, unpaired two-tailed t-test); **B)** Chat^CRE^ mice injected with Gi-coupled hM4D inhibitory floxed DREADD AAV into the basal forebrain (n=9, t(16)=1.73, p=0.104, unpaired two-tailed t-test) or **C)** the striatum (n=11, t(20)=0.067, p=0.95, unpaired two-tailed t-test) before and after CNO administration, and **D)** Chat^CRE^Cul3^F/+^ and Cul3^F/+^ mice after activation of cholinergic neurons in the basal forebrain by AAV-hM3D(Gq) DREADD injection and CNO administration (Cul3^F/+^: n=10, Chat^CRE^Cul3^F/+^: n=11, F_interaction_(1, 38)=0.69, p=0.41; F_genotype_(1, 38)=2.91, p=0.096; F_treatment_(1,38)=0.18, p=0.68, two-way ANOVA).

**Supplementary figure 2**

**
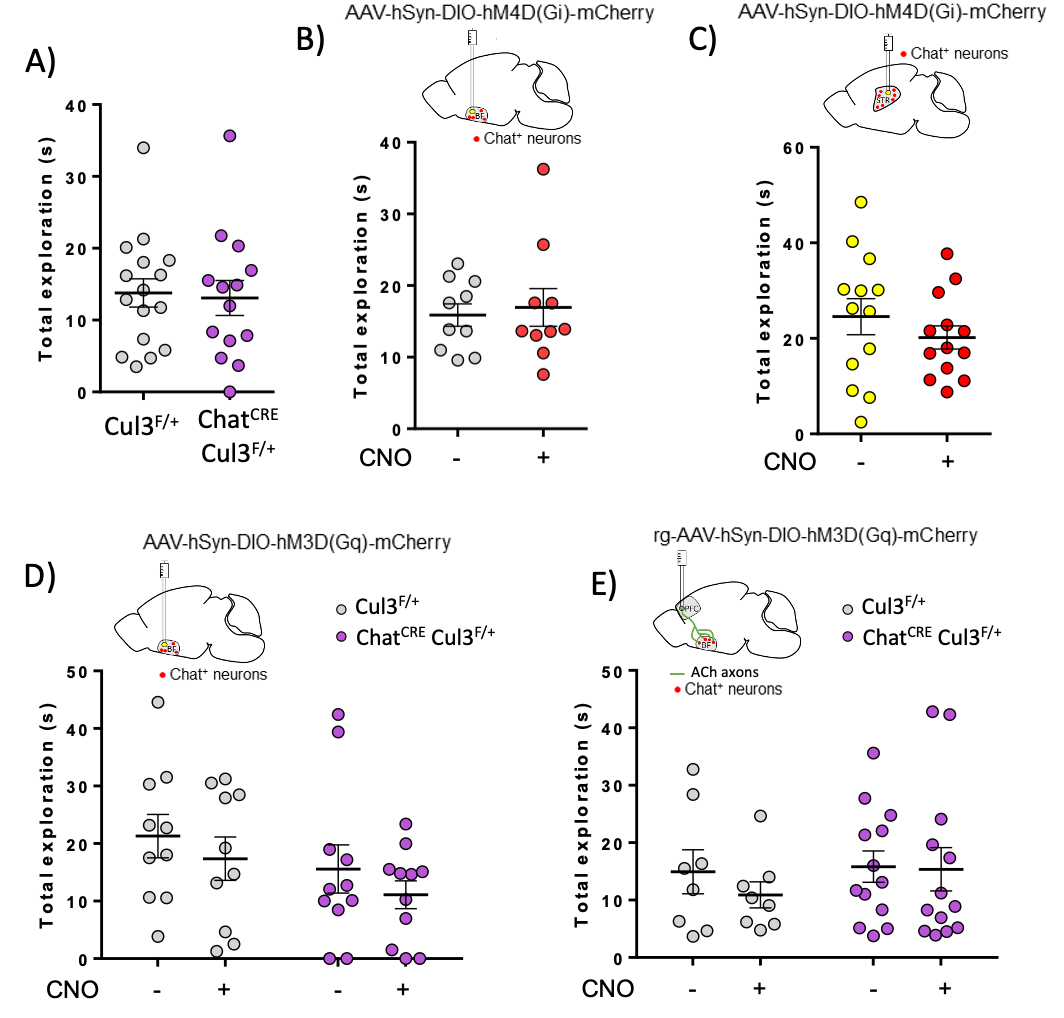
**

**Supplementary Figure 2. Total exploration time in the temporal order recognition memory (TORM) task. A)** Chat^CRE^Cul3^F/+^ and control mice displayed similar exploration despite their differences in the preference index (Cul3^F/+^: n=16, Chat^CRE^Cul3^F/+^: n=14, t(28)=0.22, p=0.83 unpaired two-tailed t-test). **B)** Stereotaxic delivery of Gi-coupled hM4D inhibitory floxed DREADD AAV into the basal forebrain of Chat^CRE^ mice did not affect total exploration after CNO administration (n=10, t(18)=0.34, p=0.74 unpaired two-tailed t-test). **C)** Unchanged exploration time after chemogenetic inactivation of striatal cholinergic neurons (n=13, t(24)=0.97, p=0.34 unpaired two-tailed t-test). **D)** Activation of BF cholinergic neurons in Chat^CRE^Cul3^F/+^ mice by AAV-hM3D(Gq) DREADD injection rescued TORM performance without significant changes in the total exploration time (Cul3^F/+^: n=10, Chat^CRE^Cul3^F/+^: n=11, F_interaction_(1, 38)=0.0056, p=0.94; F_genotype_(1, 38)=2.79, p=0.103; F_treatment_(1,38)=1.37, p=0.25, two-way ANOVA). **E)** Restoration of cognitive performance in the TORM test of Chat^CRE^Cul3^F/+^ mice by retrograde activation of cholinergic terminals in the PFC from the BF did not involve differences in exploration (Cul3^F/+^: n=8, Chat^CRE^Cul3^F/+^: n=13, F_interaction_(1, 38)=0.27, p=0.61; F_genotype_(1, 38)=0.59, p=0.44; F_treatment_(1,38)=0.42, p=0.52, two-way ANOVA).

**Supplementary figure 3**

**
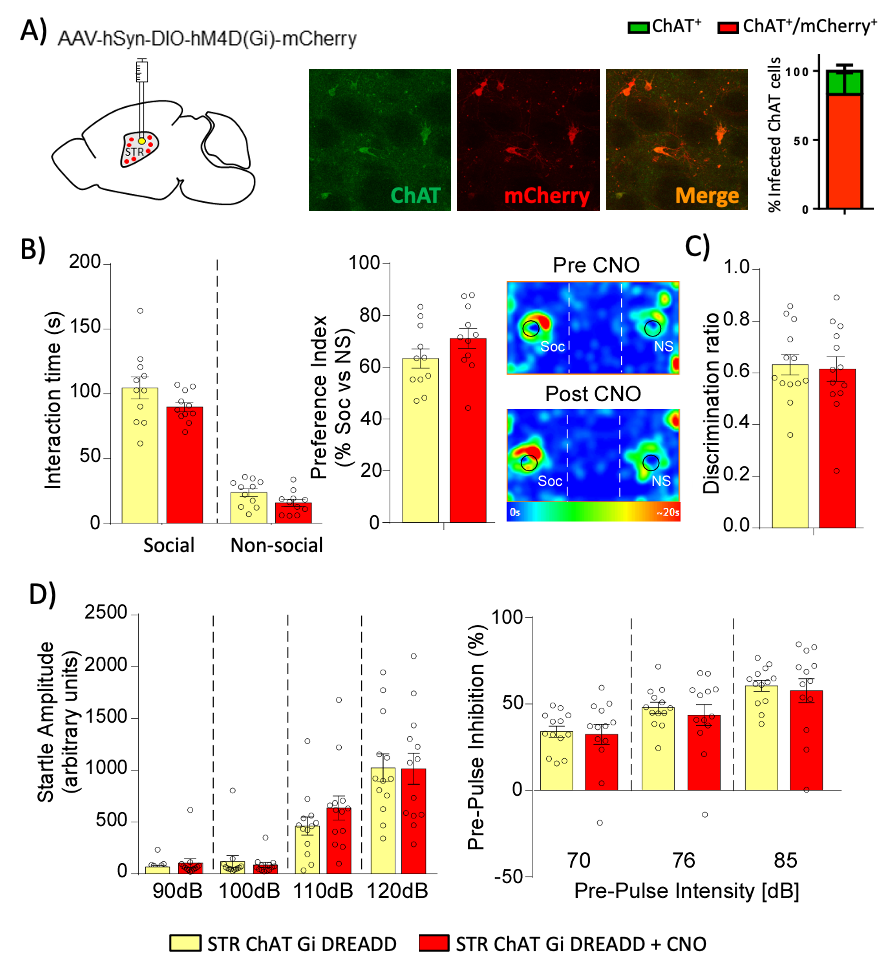
**

**Supplementary** **Figure 3. Chemogenetic inhibition of striatal cholinergic neurons in control animals does not induce behavioral changes. A)** Schematic representation of AAV-hM4D(Gi) DREADD injection into the striatum of Chat^CRE^ mice and confirmation of recombination in ~85% of cholinergic interneurons assessed in six brains. **B)** Social preference behavior is not altered after CNO administration to inhibit striatal cholinergic neurons (n=11, Interaction time: F_interaction_(1, 40)=0.45, p=0.51; F_zone_(1, 40)=238.8, p<0.0001; F_treatment_(1, 40)=5.09, p=0.03; two-way ANOVA; preference index: t(20)=1.44, p=0.17, unpaired two-tailed t-test). Inset: representative heat maps. **C)** Normal discrimination index in the temporal order recognition memory (TORM) task after CNO administration to inhibit striatal cholinergic neurons (n=13, t(24)=0.26, p=0.8, unpaired two-tailed t-test). **D)** Sensory gating is not affected by CNO administration to inhibit striatal cholinergic neurons (n=13, Startle: F_interaction_(3, 72)=0.91, p=0.44 F_stimulus_(3,72)=82.08, p<0.0001, F_treatment_(1,24)=0.18, p=0.67; PPI: F_interaction_(2, 48)=0.13, p=0.88, F_stimulus_(2,48)=43.34, p<0.0001, F_treatment_(1,24)=0.21, p=0.65, two-way RM ANOVA).

**SUPPLEMENTARY TABLE 1**

|  | SI | PPI | TORM |  |
| --- | --- | --- | --- | --- |
| Chat^CRE^Cul3^F/+^ | ↓ | ↓ | ↓ |  |
| BF ChAT neuron Gi inactivation | ↓ | = | ↓ |  |
| STR ChAT neuron Gi inactivation | = | = | = |  |
| Chat^CRE^Cul3^F/+^ + BF ChAT neuron Gq activation | = | ↑ | ↑ |  |
| Chat^CRE^Cul3^F/+^ + ChAT projection to PFC rg-Gq activation | ND | = | ↑ |  |

**Supplementary Table 1. Summary of behavioral phenotypes in the models employed in Figures 2, 4-7.** SI: social interaction, PPI: pre-pulse inhibition, TORM: temporal object recognition memory. ↓ and ↑ indicate statistically decreased and increased respectively; = represents no significant difference. ND: not determined.
